# The efficacy of paritaprevir/ritonavir/ombitasvir+dasabuvir and ledipasvir/sofosbuvir is comparable in patients who failed interferon-based treatment with first generation protease inhibitors - a multicenter cohort study

**DOI:** 10.1186/s12879-018-3465-2

**Published:** 2018-11-16

**Authors:** Ewa Janczewska, Dorota Zarębska-Michaluk, Hanna Berak, Anna Piekarska, Andrzej Gietka, Dorota Dybowska, Włodzimierz Mazur, Teresa Belica-Wdowik, Witold Dobracki, Magdalena Tudrujek-Zdunek, Zbigniew Deroń, Iwona Buczyńska, Marek Sitko, Agnieszka Czauż-Andrzejuk, Beata Lorenc, Jolanta Białkowska-Warzecha, Jolanta Citko, Łukasz Laurans, Jerzy Jaroszewicz, Łukasz Socha, Olga Tronina, Brygida Adamek, Andrzej Horban, Waldemar Halota, Barbara Baka-Ćwierz, Krzysztof Tomasiewicz, Krzysztof Simon, Aleksander Garlicki, Marta Wawrzynowicz-Syczewska, Robert Flisiak

**Affiliations:** 1 Department of Basic Medical Sciences, School of Public Health in Bytom, Medical University of Silesia, ID Clinic, Janowska 19, 41-400 Mysłowice, Bytom, Poland; 2 Department of Infectious Diseases, Wojewódzki Szpital Zespolony, Kielce, Poland; 3 Hospital of Infectious Diseases, Warszawa, Poland; 4 Department of Infectious Diseases and Hepatology, Medical University of Łódź, Łódź, Poland; 5 Department of Internal Medicine and Hepatology, Central Clinical Hospital of the MSWiA, Warszawa, Poland; 6 Department of Infectious Diseases and Hepatology, Faculty of Medicine, Collegium Medicum Bydgoszcz, Nicolaus Copernicus University Toruń, Bydgoszcz, Poland; 7 Department of Infectious Diseases, Infectious Hepatology and Acquired Immunodeficiency, Medical University of Silesia in Katowice, Chorzów, Poland; 8 Regional Center for Diagnosis and Treatment of Viral Hepatitis and Hepatology, John Paul II Hospital, Kraków, Poland; 9 MED-FIX Medical Center, Wrocław, Poland; 10 Department of Infectious Diseases, Medical University of Lublin, Lublin, Poland; 11 Ward of Infectious Diseases and Hepatology, Biegański Regional Specialist Hospital, Łódź, Poland; 12 Department of Infectious Diseases and Hepatology, Wrocław Medical University, Wrocław, Poland; 13 Department of Infectious and Tropical Diseases, Collegium Medicum, Jagiellonian University, Kraków, Poland; 14 Department of Infectious Diseases and Hepatology, Medical University of Białystok, Białystok, Poland; 15 Pomeranian Center of Infectious Diseases, Department of Infectious Diseases, Medical University of Gdansk, Gdansk, Poland; 16 Department of Infectious and Liver Diseases, Medical University of Łódź, Łódź, Poland; 17 Medical Practice of Infections, Regional Hospital, Olsztyn, Poland; 18 Department of Infectious Diseases, Hepatology and Liver Transplantation, Pomeranian Medical University, Szczecin, Poland; 19 Department of Infectious Diseases and Hepatology, Medical University of Silesia in Katowice, Bytom, Poland; 20 Department of Transplantation Medicine, Nephrology, and Internal Diseases, Medical University of Warsaw, Warszawa, Poland; 21 Warsaw Medical University & Hospital of Infectious Diseases Warszawa, Warszawa, Poland

**Keywords:** Chronic hepatitis C, Liver cirrhosis, Protease inhibitors, Retreatment, Sustained virologic response

## Abstract

**Background:**

According to the EASL and AASLD guidelines, the recommended treatment for patients who failed to achieve a sustained virologic response (SVR) on prior interferon-based triple therapy with protease inhibitors (PI), is a combination of sofosbuvir and NS5A inhibitors. Polish national recommendations also allow the use of paritaprevir/ritonavir/ombitasvir+dasasbuvir±ribavirin (PrODR) in this group of patients. The aim of the study was to evaluate the efficacy and safety of PrODR vs. ledipasvir/sofosbuvir±RBV (LSR) in PI-experienced patients in real-life setting.

**Methods:**

Our analysis included patients registered in the nationwide, investigators initiated, multicentre EpiTer-2 database.

Among 4530 patients registered, 335 with genotype 1 (93% 1b) were previously treated with IFN-based regimens with PIs: 127 with boceprevir (BOC), 208 with telaprevir (TVR).

Patients with advanced fibrosis (F3/F4) were significantly predominant (BOC 28.4%/61.4%, TVR 18.8%/64.4%, respectively).

Subjects were assigned to IFN-free retreatment as follows: BOC - 64 (50.4%) PrODR and 63 (49.6%) LSR; TVR- 103 (49.5%) PrODR and 105 (50.5%) LSR.

**Results:**

SVR rates were comparable for particular groups: BOC → PrODR- 100%; BOC → LSR - 98%; TVR → PrODR - 97%; TVR → LSR - 96% (intent-to treat analysis-ITT) and BOC → PrODR→100%; BOC → LSR - 99%; TVR → PrODR - 99%; TVR → LSR - 98% (modified intent-to treat analysis-mITT).

Both treatment regimens had a favourable safety profile. Adverse events (AEs) were generally mild or moderate in severity. Three deaths were reported. The treatment was stopped due to AEs in five patients (three treated with PrODR and two with LSR).

**Conclusion:**

Efficacy and safety of treatment with PrODR and LSR is comparable in BOC or TVR-experienced patients.

## Background

Progress achieved in recent years in the treatment of patients with viral hepatitis C has enabled elimination of hepatitis C virus (HCV) infection in most patients. This progress has been achieved through the use of drugs that produce a direct antiviral action (direct-acting antivirals-DAAs). These therapies are highly effective even in patients with advanced fibrosis, as well as hepatic insufficiency. Effective therapy inhibits progression of the disease, often leading to a fibrosis regression [1–4].

The efficacy of DAA-based therapies can be reduced by the presence of substitutions causing drug resistance (resistance-associated substitutions-RASs) [5–7]. Such substitutions can occur in patients untreated previously; however, their occurrence is more often associated with ineffective antiviral therapy, which involved DAAs with a low genetic barrier, for example first-generation protease inhibitors (PI) such as boceprevir (BOC) or telaprevir (TVR).

These drugs were the first DAAs used with pegylated interferon (PegIFN) and ribavirin (RBV) in antiviral therapies for patients infected with HCV genotype 1.

In the following years, drugs belonging to other classes and having other mechanism of action disrupting the process of HCV replication were introduced: polymerase inhibitors and non-structural protein 5A (NS5A) inhibitors. The combined use of drugs belonging to 2 or 3 therapeutic groups allowed the development of effective and safe interferon-free regimens.

In patients who had already undergone ineffective BOC or TVR triple therapy, there was a risk of reduced efficacy of subsequent IFN-free therapies in which one of the components was a protease inhibitor due to the RASs generated during the first use of these drugs.

According to the guidelines of the European Association for the Study of the Liver (EASL), and the American Association for the Study of the Liver Diseases (AASLD), the re-use of first-generation protease inhibitors is not recommended in patients who do not respond to these drugs in the past [8, 9].

Recommended therapeutic regimens were combinations of polymerase and NS5A inhibitors: ledipasvir/sofosbuvir or daclatasvir+sofosbuvir [8, 9]. These recommendations were based on the randomized clinical trials findings [10–13]. Moreover, in this patient group, the EASL recommendations provide for ribavirin addition to the DAAs to improve efficacy and reduce potential resistance [8].

In Poland, the first and for some time the only therapy composed of DAAs was paritaprevir/ ritonavir/ombitasvir ± dasabuvir ± ribavirin (PrODR), not mentioned in the above guidelines as recommended for patients after PI treatment failure.

Initially, this drug combination was used in the early access program, in patients with advanced fibrosis, the majority of whom underwent ineffective prior IFN-based treatment (AMBER study) [14]. Among the patients included in this cohort were those who failed triple therapies involving boceprevir or telaprevir. However, the number of these patients was small. High efficacy observed in this group were considered in the recommendations of the Polish Group of HCV Experts [15] and caused inclusion of PrODR along with ledipasvir/sofosbuvir ±RBV (LSR) and sofosbuvir + daclatasvir (SOF + DCV), to be used in patients with a history of prior BOC + PegIFN+RBV or TVR + PegIFN+RBV regimens.

In 2015, PrODR and LSR became available (reimbursed) for Polish patients, whereas SOF + DCV combination was not accepted for reimbursement in Poland.

The purpose of this study was to evaluate in the real-life setting the efficacy and safety of PrODR versus LSR in patients who failed prior triple IFN-based therapies with first generation protease inhibitors.

## Material and methods

### Study population

On the investigators’ initiative, a national EpiTer-2 database of patients receiving antiviral treatment due to HCV infection in Poland was established in 2016 based on regimens available within the therapeutic program of the National Health Fund. Twenty-two hepatology centres applied for participation in the project. Treatment efficacy and safety data were collected in the EpiTer-2 web database.

Demographic data of the patients, information related to HCV genotype, stage of fibrosis, liver function parameters (Child-Turcotte-Pugh and MELD scores), prior antiviral therapy, concomitant diseases and drugs used in relation thereto, HBV and/or HIV coinfections were collected in the database.

Hepatic fibrosis was evaluated by liver biopsy based on the METAVIR or Scheuer scoring system, transient elastography (TE) using the FibroScan (Echosens, Paris) device or the real-time shear wave elastography (SWE) using the Aixplorer (Supersonic, Aix-en-Provence) device. Among BOC-experienced patients: liver biopsy was performed in 31 (24.4%), TE in 73 (57.5%), and SWE in 23 (18.1%) patients, and in the TVR group: biopsy was performed in 44 (21.2%), TE in 138 (66.3%), and SWE in 26 (12.5%) patients.

HCV RNA was monitored prior to and after the treatment (end of treatment virologic response: EOT-VR), and then after at least 12-week follow-up period (sustained virologic response -SVR). Two assays were used to measure HCV RNA, depending on local practices at the testing site: Roche COBAS TaqMan with a lower limit of quantification (LLOQ) of 15 IU/mL or Abbott RealTime with an LLOQ of 12 IU/mL.

Adverse events (AEs) observed during the treatment and follow-up period were reported as well. Criteria for assessing AEs as serious were: resulting in death, life-threatening, requiring hospitalisation or prolongation of existing hospitalization, resulting in persistent or significant disability, or congenital anomaly or birth defect.

In 2016 and 2017 up to now, a total of 4530 patients were registered in the EpiTer-2 database, including 335 patients having failed prior triple-drug regimens with boceprevir or telaprevir, retreated with interferon-free regimens PrOD and LSR, being the subject of this study.

Baseline characteristics of the patients are presented in Table 1. Sex, age, and BMI distribution were similar for both BOC- and TVR-experienced groups of patients. Notably, patients with GT1b prevailed significantly in both groups, which is typical for Polish population [16]. Patients with cirrhosis (F4) and advanced fibrosis (F3) were predominant, while only few patients showed severe liver impairment symptoms (Child-Turcotte-Pugh B or C) at the beginning of the therapy or decompensation in the previous history. The percentage of patients with confirmed oesophageal varices was similar in both subgroups.

**Table 1:** Baseline Characteristics of 335 Patients Included in the Study

Parameter	Boceprevir-experienced	Telaprevir-experienced
Number of patients, n (%)	127 (38%)	208 (62%)
Gender: females/males, n (%)	63 (49.6%)/65 (50.4%)	95 (45.7%)/ 113 (54.3%)
Age (years) mean ± SD; min-max	55.4 ± 10.9; 23–74	60.3 ± 10.7; 27–78
BMI mean ± SD; min-max	27.4 ± 4.99; 19–38	27.6 ± 4.11; 19–44
HCV Genotype: n (%)		
1b	120 (94.5%)	191 (91.8%)
1a	5 (3.9%)	9 (4.3%)
1	2 (1.6%)	8 (3.9%)
Fibrosis, n (%)		
F4	78 (61.4%)	134 (64.4%)
F3	36 (28.4%)	39 (18.8%)
F2	9 (7.1%)	19 (9.1%)
F1	4 (3.1%)	16 (7.7%)
Child-Turcotte-Pugh, n (%)		
A	123 (96.9%)	201 (96.6%)
B	4 (3.1%)	5 (2.4%)
C	0 (0%)	2 (1%)
Response to previous treatment with PI+PegIFN+RBV, n (%)		
Non-response	45 (35.4%)	83 (39.9%)
Relapse	48 (37.8%)	66 (31.7%)
Discontinuation	17 (13.4%)	37 (17.8%)
Unknown	17 (13.4%)	22 (10.6%)
History of hepatic decompensation, n (%)	9 (7.1%)	11 (5.3%)
Ascites	9 (7.1%)	9 (4.3%)
Encephalopathy	0 (0%)	2 (1%)
Documented oesophageal varices, n (%)	21 (16.5%)	47 (22.6%)
History of hepatocellular carcinoma, n (%)	1 (0.8%)	4 (1.9%)
HBV coinfection, n (%)		
HBsAg positive	4 (3.1%)	0 (0%)
HBV DNA positive	0 (0%)	0 (0%)
Anti-HBc positive	11 (8.7%)	17 (8.2%)
HIV coinfection, n (%)	0 (0%)	0 (0%)
History of liver transplantation, n (%)	1 (0.8%)	5 (2.4%)
Comorbidities, n (%)		
Any comorbidity	87 (68.5%)	142 (68.3%)
Hypertension	48 (37.8%)	82 (39.4%)
Diabetes	17 (13.4%)	34 (16.3%)
Renal insufficiency	1 (0.8%)	2 (1%)
Autoimmune diseases	2 (1.6%)	2 (1%)
Non-HCC tumours	1 (0.8%)	0 (0%)
Other	21 (16.5%)	46 (22.1%)
Concomitant medications, n (%)	81 (63.8%)	141 (67.8%)

The choice of the drug, dosage and length of treatment regimen (12 vs. 24 weeks, addition of ribavirin) was made by the treating physicians based on the applicable product characteristics and recommendations of Polish Group of HCV Experts [15, 17]. All patients qualified for class B or C of Child-Turcotte-Pugh scoring system received LSR.

### Statistical analysis

Data are presented as absolute numbers (%) or mean ± standard deviation. No sample size was planned. All patients who started the treatment were included in the analysis, and efficacy analyses were performed on an intent-to-treat (ITT) basis (missing virological measurements were imputed as treatment failures) and modified intent-to-treat (mITT) basis, which excludes patients with missing data of sustained virologic response (at least 12 weeks after treatment completion). The proportion of patients who achieved SVR was calculated. The significance of difference was calculated by use of Chi-square or Fischer’s exact test where appropriate. *P* values of < 0.05 were considered to be statistically significant.

Statistical analyses were performed with STATISTICA12.0 (Statsoft, Tulsa, OK, USA).

## Results

This study focuses on the analysis of the data from the EpiTer-2 database concerning 335 patients having failed prior triple, IFN-based regimens with boceprevir or telaprevir. Patients previously treated with telaprevir containing regimen prevailed (62%) in the cohort (Table 1).

Patients with relapse of infection following prior antiviral treatment prevailed in BOC subgroup, while those with non-response prevailed in TVR subgroup. There were no patients with HIV coinfection or active HBV coinfection. Only a few patients had a history of hepatocellular carcinoma (one in BOC and four in the TVR subgroup). Moreover, in both subgroups, there were patients who began treatment after liver transplantation (one in BOC and five in the TVR subgroup).

A majority of patients from both subgroups had concomitant diseases, most often hypertension and diabetes. BOC patients (63.8%) and 67.8% of TVR patients were taking additional drugs due to these diseases.

Different dosage regimens of PrODR or LSR were applied in this therapy depending on the current guidelines. Among 127 BOC patients 64 (50.4%) were being treated using one of the PrODR (BOC➔PrODR), while 63 (49.6%) patients with LSR regimens (BOC➔LSR). In the TVR subgroup, 103 (49.5%) patients received PrODR (TVR➔PrODR) and 105 (50.5%) patients received LSR (TVR➔LSR). Distribution of these regimens among studied population is summarized in Table 2.

**Table 2:** Current Treatment Regimens

Treatment regimen	Boceprevir-experienced	Telaprevir-experienced
LDV/SOF, n (%)	*n* = 63	*n* = 105
LDV/SOF 12 weeks	11 (8.7%)	26 (12.5%)
LDV/SOF 24 weeks	6 (4.7%)	9 (4.3%)
LDV/SOF + RBV 12 weeks	41 (32.3%)	63 (30.3%)
LDV/SOF + RBV 24 weeks	5 (3.9%)	7 (3.4%)
PrOD, n (%)	*n* = 64	*n* = 103
PrOD 12 weeks	29 (22.8%)	42 (20.2%)
PrOD+RBV 12 weeks	33 (26%)	56 (26.9%)
PrOD+RBV 24 weeks	2 (1.6%)	5 (2.4%)

### Treatment efficacy

**Fig. 1:**
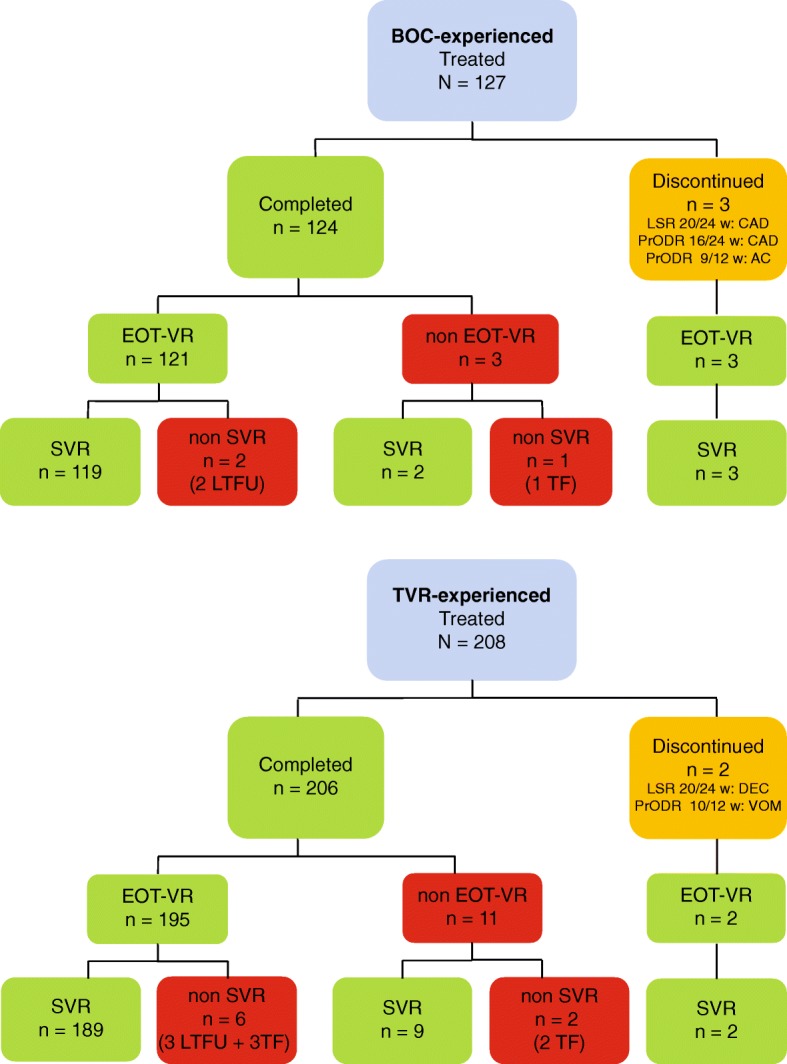
Patients’ disposition and reasons for discontinuation. *EOT-VR* end of treatment virologic response, *SVR* sustained virologic response, *LTFU* lost to follow-up, *TF* treatment failure, *DEC* hepatic decompensation, *CAD* exacerbation of pre-existing coronary arterial disease, *AC* acute cholecystitis, VOM-vomiting

Figure 1 presents the treatment course and the reasons of discontinuation.

In both groups, a majority of patients completed the full course of treatment scheduled and only five patients (1.5%) had to interrupt it due to adverse events (AEs). All patients, whose treatment was discontinued, reached SVR despite the reduced duration of treatment. Only one BOC and two TVR patients (non-responders) failed to achieve SVR among the total number of 14 patients in whom HCV RNA was detectable at the end of the treatment (EOT).

The remaining subjects achieved SVR despite a positive result at the EOT. This phenomenon, specific for DAA regimens, was not observed during interferon-based therapies.

On the other hand, despite of undetectable HCV RNA at the EOT, 3 patients relapsed. Two patients from BOC and three from TVR group were lost to follow-up and evaluation of SVR was not possible.

**Fig. 2:**
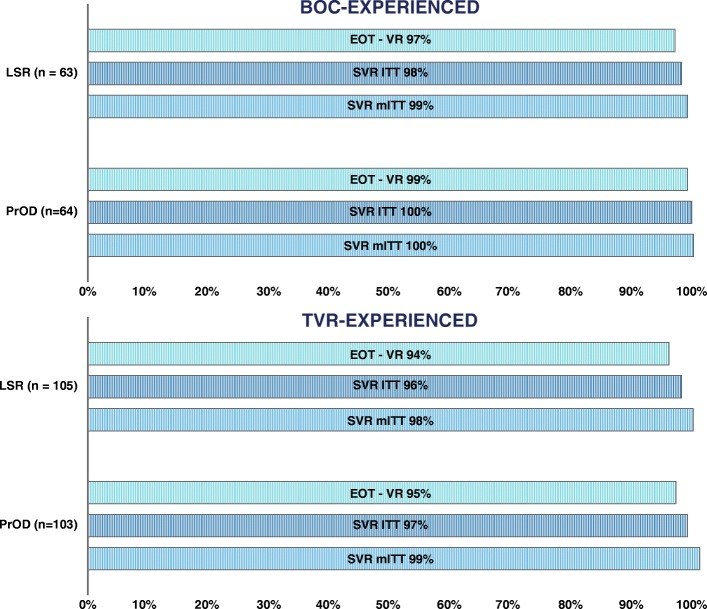
Treatment outcome. End of treatment virologic response (EOT-VR) and sustained virologic response (SVR) rate; ITT—intent-to-treat analysis, which included all patients receiving at least 1 dose of the treatment, mITT—modified ITT analysis, which excluded patients with missing data of sustained virologic response (at least 12 weeks after treatment completion)

As shown in Fig. 2, end of treatment virologic response (EOT-VR) and SVR evaluated in the ITT analysis was insignificantly lower in TVR groups.

In the mITT evaluation in BOC group, SVR was 99% for BOC➔LSR and 100% for BOC➔PrODR, while in TVR group it was 98% for TVR➔LSR and 99% for TVR➔PrODR.

Therefore, we can assume that both drugs are highly effective irrespective of the first-generation protease inhibitor which was used in the primary treatment.

### Treatment safety

The most important adverse events (AEs) that occurred during the treatment and in the follow-up period are summarized in Table 3.

**Table 3:** Adverse Events

Parameter	Boceprevir-experienced		Telaprevir-experienced	
	LDV/SOF (*n* = 63)	PrOD (*n* = 64)	LDV/SOF (*n* = 105)	PrOD (*n* = 103)
Patients with at least one AE, n (%)	24 (38.1%)	28 (43,8%)	44 (41.9%)	45 (43.7%)
RBV-containing regimen	18 (28.6%)	19 (29.7%)	31 (29.5%)	33 (32%)
Most common AEs (> 5%), n (%)				
Fatigue	15 (23.8%)	17 (26.6%)	27 (25.7%)	30 (29.1%)
Anaemia	9 (14.3%)	13 (20.3%)	15 (14.3%)	22 (21.4%)
Headache	8 (12.7%)	11 (17.2%)	10 (9.5%)	12 (11.7%)
Nausea	4 (6.3%)	4 (6.3%)	7 (6.7%)	8 (7.8%)
Serious AEs, n (%)	1 (1.6%)	1 (1.6%)	2 (1.9%)	1 (1%)
Deaths	1 (1.6%)	0 (0%)	2 (1.9%)	0 (0%)

These were observed in 52 patients of BOC group and in 99 patients of TVR group. Severity was generally mild or moderate. Fatigue, headache, and anaemia prevailed, which mainly occurred in the patients treated with therapeutic regimens containing ribavirin. Treatment discontinuation due to AE occurred in three patients of the BOC group and two patients of the TVR group (Fig. 1).

According to the treating physicians’ evaluation, AEs leading to discontinuation of the treatment in BOC group, were not associated with the DAA applied, while persistent vomiting in one TVR patient was probably associated with PrODR.

The second case (decompensation) was probably associated with the baseline stage of the disease rather than antiviral treatment. It occurred in a patient with a history of hepatic insufficiency treated with LSR.

Serious adverse events (SAEs) occurred in two patients from BOC group. One of them, being treated with PrODR, underwent cholecystectomy due to acute cholecystitis, which entailed discontinuation of the treatment on the 9th week. Another patient was diagnosed with cholangiocarcinoma after completing treatment with LSR and died on the 20th week of the follow-up period.

In TVR group, two patients treated with LSR died of cancer during the follow-up period (hepatocellular carcinoma and pancreatic cancer). Both patients had undetectable HCV RNA 12 weeks after the treatment end. Portal vein thrombosis was detected in one patient treated with PrOD in the course of the treatment. This patient also achieved SVR.

All SAEs were assessed as irrelevant to the antiviral treatment and at least SVR12 was confirmed in all of these patients.

## Discussion

The introduction of interferon-free therapy regimens had a beneficial effect in patients with chronic hepatitis C. These regimens have become highly effective and safe, irrespective of the severity of liver disease.

In the interferon era, prior treatment failure was an important factor restricting efficacy of subsequent antiviral therapies [18–20]. Introduction of triple-drug regimens significantly increased chances for recovery only in patients with mild to moderate fibrosis, treatment naïve or relapsers after PegIFN+RBV therapy. Patients with cirrhosis and/or lack of virologic response to prior therapy showed significantly worse response to the interferon-based treatment, which included first generation PIs [21–25].

Breakthrough was only made through the use of interferon-free therapies. As specified in the introduction, international hepatology societies recommend the use of LSR or SOF + DVC after failed triple-drug therapies. Other available drugs (simeprevir+SOF, PrOD) were not recommended for this patient group [8, 9]. However, daily clinical practice and local conditions do not always enable rigorous compliance with these guidelines. Combinations of SOF with NS5A inhibitor was not available in Poland until November 2015. The first interferon-free drug which could be used was PrODR, initially available under the early-access program and, beginning from July 2015, under the program of the National Health Fund.

Due to the urgent need to apply the therapy to numerous queuing patients with advanced fibrosis and cirrhosis, they were given an accessible drug, which was considered a rescue therapy. The first group of patients (AMBER cohort) also included 16 patients who had failed triple treatment with boceprevir or telaprevir [14]. SVR was achieved in all these patients. Taking into account these encouraging results and local realities, the Polish Group of HCV Experts included this therapeutic regimen in its recommendations as acceptable for use in the patients previously treated by PIs. This drug was approved by the national health insurance institution and started to be widely used within the framework of the drug program, first as the only drug and then along with LSR.

Therefore, the Epi-Ter2 program enabled to gather a vast group of patients treated with the PrODR regimen seldom used in other countries.

Description of small patient groups can be mostly found in the literature, e.g. only 7 patients after PI treatment, who received PrODR therapy, were included in the Italian VIRONET-C cohort [26].

A large German cohort involving a total of 1017 patients treated by PrODR [27], included 72 patients treated previously with TVR or BOC. A Spanish cohort consisting of 1567 patients included only 49 patients treated by PrODR [28]. In the aforementioned cohorts, most of the patients with the history of PI therapy, received LSR. Therefore, our patient group is one of the largest, in which efficacy of PrODR in patients after first generation PI treatment was evaluated.

In our study, we demonstrated that PrODR regimens are as effective in this patient group as LSR, including patients with advanced liver disease irrespective of prior treatment failure.

Initial concerns were not substantiated that first-generation PI treatment would be associated with selection of RASs significantly reducing efficacy of subsequent therapies. Any probable RAS faded, and wild virus type begins dominating within the period of a year after the end of the PI therapy. Whereas, RASs in NS5A region characterized by higher durability had a demonstrable influence on subsequent treatment efficacy, which proved to be a significant problem [29–31].

The baseline presence of RAS in our patients was not tested because it is not a routine examination in real-life setting. For the majority of patients, the period between the end of the triple drug treatment and the start of the interferon-free treatment was longer than 12 months.

Both therapy types were characterized by a favourable safety profile, including cirrhosis patients.

The strength of our study is a large group of patients treated with the therapeutic regimen, which is relatively infrequently used in the patients after PI in Western Europe and USA. Results of our study confirm that PrODR can be used successfully and equivalently with other accessible drugs in patients with compensated liver disease. This can be particularly relevant for countries where the full scope of the interferon-free therapies is unavailable, or administrative restrictions in the choice of drugs exist.

The limitation may be the use of different therapeutic regimens in individual subgroups of patients (duration of treatment, addition of RBV) which results from the fact that the study was conducted in real-life conditions, without a predetermined protocol. However, all the therapeutic regimens, used according to the current label, were characterized by very high efficacy.

## Conclusion

In the future, as availability increases, novel, pangenotypic DAAs will surely start dominating. However, as long as an access to these newest drugs is limited, the drugs of the previous generation will remain in widespread use.

The results of our study may be useful in the daily clinical practice, proving that the patients after failed IFN-based triple therapies containing PI, can be cured using both PrODR and LSR regimens with comparable efficacy and safety.

## Abbreviations

AASLD: American European Association for the Study of the Liver Diseases
AE: Adverse Adverse event
BOC: BoceprevirBoceprevir
DAAs: DirectDirect-acting antivirals
DCV: DaclatasvirDaclatasvir
EASL: European Association for the Study of the Liver
EOT-VR: End End of treatment virologic response
HBV: Hepatitis Hepatitis B virus
HCV: Hepatitis Hepatitis C virus
HIV: Human Human immunodeficiency virus
ITT: IntentIntent-to treat analysis
LSR: LedipasvirLedipasvir/sofosbuvir ± RBV
mITT: modified intent-to treat analysis
NS5A: NonNon-structural protein 5A
PegIFN: Pegylated Pegylated interferon
PI: Protease Protease inhibitor
PrODR: ParitaprevirParitaprevir/ritonavir/ombitasvir+dasasbuvir ±ribavirin
RASs: ResistanceResistance-associated substitutions
RBV: RibavirinRibavirin
SAE: Serious Serious adverse events
SOF: SofosbuvirSofosbuvir
SVR: Sustained Sustained virologic response
SWE: Shear Shear wave elastography
TE: Transient Transient elastography
TVR: TelaprevirTelaprevir
